# Horizontally Acquired Biosynthesis Genes Boost *Coxiella burnetii*'s Physiology

**DOI:** 10.3389/fcimb.2017.00174

**Published:** 2017-05-10

**Authors:** Abraham S. Moses, Jess A. Millar, Matteo Bonazzi, Paul A. Beare, Rahul Raghavan

**Affiliations:** ^1^Department of Biology and Center for Life in Extreme Environments, Portland State UniversityPortland, OR, USA; ^2^Centre National de la Recherche Scientifique, Formation de Recherche en Évolution 3689, Centre d'Études d'Agents Pathogènes et Biotechnologies Pour la Santé, Université MontpellierMontpellier, France; ^3^Coxiella Pathogenesis Section, Laboratory of Bacteriology, Rocky Mountain Laboratories, National Institutes of HealthHamilton, MT, USA

**Keywords:** *Coxiella burnetii*, lateral gene transfer, heme biosynthesis, biotin biosynthesis, LPS, glutamic acid, horizontal gene transfer (HGT)

## Abstract

*Coxiella burnetii*, the etiologic agent of acute Q fever and chronic endocarditis, has a unique biphasic life cycle, which includes a metabolically active intracellular form that occupies a large lysosome-derived acidic vacuole. *C. burnetii* is the only bacterium known to thrive within such an hostile intracellular niche, and this ability is fundamental to its pathogenicity; however, very little is known about genes that facilitate *Coxiella*'s intracellular growth. Recent studies indicate that *C. burnetii* evolved from a tick-associated ancestor and that the metabolic capabilities of *C. burnetii* are different from that of *Coxiella*-like bacteria found in ticks. Horizontally acquired genes that allow *C. burnetii* to infect and grow within mammalian cells likely facilitated the host shift; however, because of its obligate intracellular replication, *C. burnetii* would have lost most genes that have been rendered redundant due to the availability of metabolites within the host cell. Based on these observations, we reasoned that horizontally derived biosynthetic genes that have been retained in the reduced genome of *C. burnetii* are ideal candidates to begin to uncover its intracellular metabolic requirements. Our analyses identified a large number of putative foreign-origin genes in *C. burnetii*, including tRNA^Glu^2 that is potentially required for heme biosynthesis, and genes involved in the production of lipopolysaccharide—a virulence factor, and of critical metabolites such as fatty acids and biotin. In comparison to wild-type *C. burnetii*, a strain that lacks tRNA^Glu^2 exhibited reduced growth, indicating its importance to *Coxiella*'s physiology. Additionally, by using chemical agents that block heme and biotin biosyntheses, we show that these pathways are promising targets for the development of new anti-*Coxiella* therapies.

## Introduction

*Coxiella burnetii* is the etiological agent of acute Q fever and a chronic disease commonly manifested as endocarditis (Maurin and Raoult, 1999). Most human infections occur through inhalation of aerosols originating from ruminants that shed *C. burnetii* during parturition and in milk. The pathogen persists in the environment as a metabolically quiescent small cell variant (SCV), which transforms into a metabolically active large cell variant (LCV) within a lysosome-derived, acidic (pH ~4.5), *Coxiella*-containing vacuole (CCV) (Voth and Heinzen, 2007). The unique ability of *C. burnetii* to thrive in this inhospitable vacuole is fundamental to its physiology and pathogenicity; however, the metabolic processes that drive its intracellular growth are unknown.

The evolutionary origin of *C. burnetii* is also not clearly understood. The closest relatives of *C. burnetii* are tick-associated bacteria, indicating that *C. burnetii* evolved from a tick-associated ancestor (Duron et al., 2015; Gottlieb et al., 2015; Smith et al., 2015). Interestingly, *Coxiella*-like bacteria found in ticks cannot infect mammalian cells and are unable to grow in ACCM-2, a culture medium that supports robust growth of *C. burnetii* (Omsland et al., 2011; Duron et al., 2015). These observations suggest that despite their close evolutionary relationship, the human pathogen, and the tick-associated strains have different virulence and metabolic capabilities. Concomitantly, when the genomes of *C. burnetii* were sequenced, it became clear that the pathogen has acquired several virulence and metabolic genes via horizontal gene transfer (HGT). For example, *C. burnetii* contains a tryptophan biosynthesis operon of Chlamydial origin, and a Type Four Secretion System (TFSS) and eukaryote-like ankyrin repeat sequence-containing effector proteins that are essential for CCV generation (Seshadri et al., 2003; Beare et al., 2009). Horizontal acquisition of foreign DNA occurs in bacteria through transformation, conjugation, or transduction via mobile genetic elements such as plasmids, integrons, bacteriophages, transposons, retrotransposons etc. The novel DNA is stably maintained and spreads through the recipient population if it offers selective advantage (e.g., antibiotic resistance), allowing the bacterium to adapt to the new environment (Eisen, 2000; Frost et al., 2005; Thomas and Nielsen, 2005).

Unlike eukaryotic genomes, which contain large fractions of non-functional DNA (e.g., >80% of human genome), bacterial genomes are tightly packed with functional genes (Moran, 2002; Ochman and Davalos, 2006). In bacteria there is a bias toward deletion over insertion, hence, DNA is retained in a bacterial genome only if selection is acting effectively to preserve it (Mira et al., 2001). For instance, when the tick-associated ancestor of *C. burnetii* evolved into a mammalian pathogen that replicates only within the CCV, several biosynthetic genes would be rendered redundant if corresponding metabolic intermediates are available within the host cell. In addition, although genes acquired via HGT might have been critical in facilitating the host shift, many of them could become expendable in the new lifestyle (Lo et al., 2015). These superfluous metabolic and HGT-origin genes would subsequently be deleted from the genome due to a lack of selection pressure to maintain them (Ochman and Moran, 2001). Furthermore, the intracellular niche limits *C. burnetii*'s opportunity to gain new genes from the environment via HGT. Thus, intracellular pathogens such as *C. burnetii* tend to have reduced genomes in comparison to related free-living bacteria (e.g., *C. burnetii*'s genome is ~2 million bp, whereas *E. coli*'s is ~5 million bp). Based on these observations, we reasoned that a significant number of HGT-origin genes that have been retained in *C. burnetii* would be critical to its intracellular fitness. In this study, we identified a large number of horizontally derived genes, including those for the synthesis of LPS, fatty acids, heme, and biotin that augment the physiological capability of *C. burnetii*.

## Results and discussion

### Identification of horizontally acquired genes in *C. burnetii*

**Figure 1:**
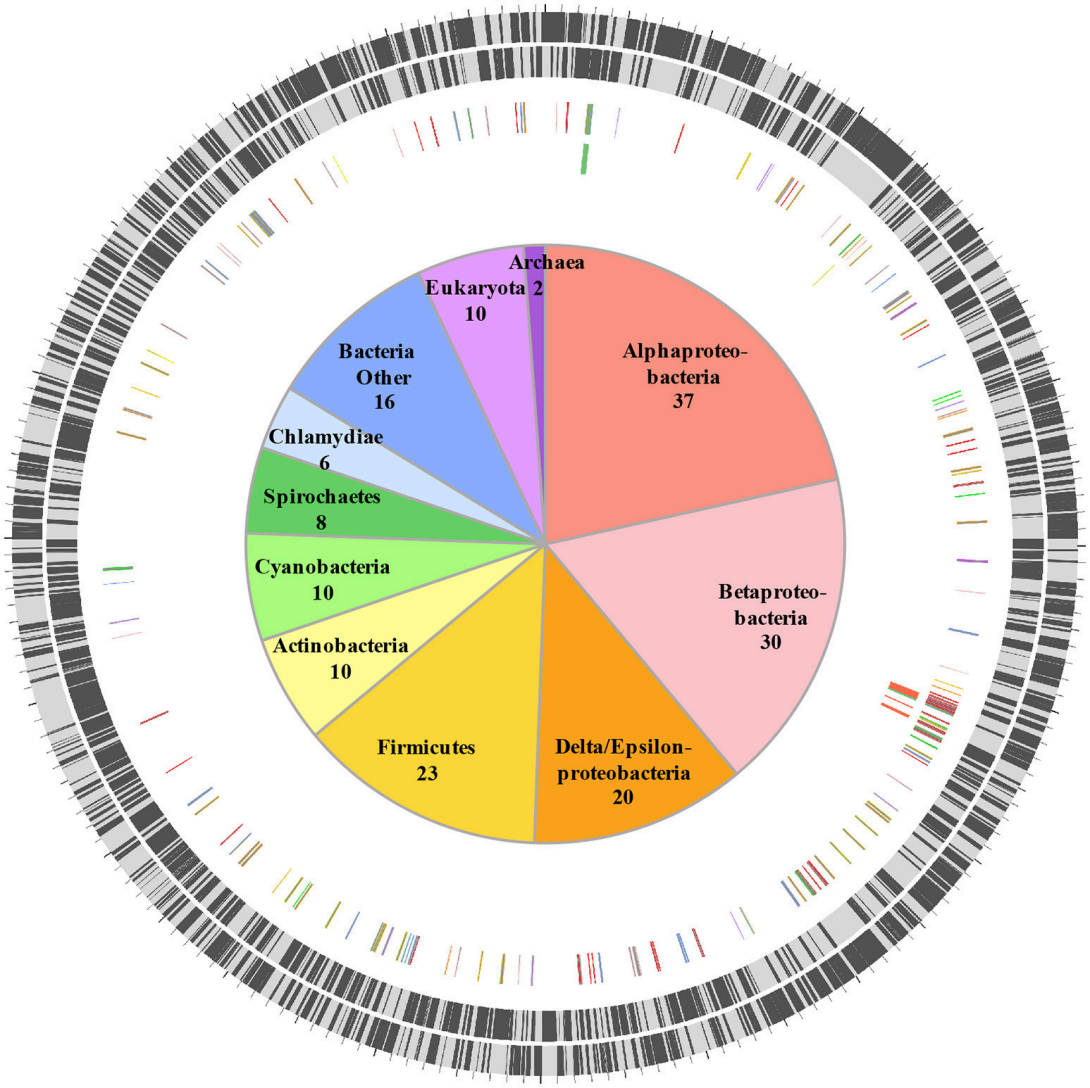
**Horizontally acquired genes in ***C. burnetii*****. Two outer rings show ORFs (black bars) on forward and reverse strands, respectively. Third ring shows positions of 172 HGT-origin genes, and the inner ring contains biosynthetic genes examined in this study. Genes are colored according to their putative donors, as shown in the center. Number of genes acquired from each donor is also shown.

HGT is a major driver of evolution and adaptation in bacteria (Lerat et al., 2005; Price et al., 2008; Treangen and Rocha, 2011). By examining differences in nucleotide composition (e.g., GC%) HGT-origin genes can be tentatively identified; however, because most transfers occur between closely related bacteria, and because genes from distant organisms will evolve over time to reflect the base composition of the recipient genome (Lawrence and Ochman, 1997), this approach is not always effective. Another common approach is to perform reciprocal-BLAST analysis (e.g., Raghavan et al., 2012), where, if the top hit is from a distantly related organism, the gene could be of putative HGT origin. While this approach provides rapid results, the best BLAST hit may not always be the closest phylogenetic relative of the query gene (Koski and Golding, 2001). This is especially the case for organisms such as *C. burnetii* that have few close relatives represented in NCBI databases. For instance, *C. burnetii* is the only defined species within its genus, and complete genomes are available for only three other bacteria within the order Legionellales—*Legionella, Rickettsiella*, and *Diplorickettsia*. In order to circumvent this limitation, we utilized a phylogeny informed BLAST-based approach (HGTector, Zhu et al., 2014), but applied very strict criteria to categorize a gene as horizontally acquired. We set *Coxiella* as the self-group, and Legionellales as the exclusion group, which captured HGT events where only *Coxiella* has acquired a particular gene from outside of Legionellales and ignored any events where the genes could also have been transferred elsewhere within the order. In addition, because the phylogenetic position of Legionellales within Gammaproteobacteria is not well-resolved (Williams et al., 2010), we also ignored any gene that was potentially gained from another member of the class Gammaproteobacteria. Using this ultra-conservative approach, we were able to identify 172 “high-confidence” horizontally acquired genes on the chromosome of *C. burnetii* RSA 493 (Figure 1, Dataset S1), whereas, none of the genes located on *C. burnetii* plasmids (QpH1, QpRS, QpDG) were deemed to be of horizontal origin.

Orthologs of all 172 HGT-origin genes are present in *C. burnetii* Dugway 5J108-111 (NC_009727.1), *C. burnetii* 3262 (CP013667.1), and *C. burnetii* Z3055 (NZ_LK937696.1), but we couldn't detect orthologs of CBU_1991 in *C. burnetii* RSA 331 (CP000890.1), CBU_2021 and CBU_0562 in *C. burnetii* G_Q212 (NC_011527.1), and CBU_0007a, CBU_0167, CBU_0168, CBU_0768, and CBU_0792 in *C. burnetii* K_Q154 (NC_011528.1). Of the 172 HGT genes, 18 are located close to transposase genes in *C. burnetii* RSA 493, suggestive of a role for transposons or insertion sequences in acquiring foreign genes. In addition to the large number of transposases (>30), all *C. burnetii* strains contain several other selfish genetic elements that are horizontally exchanged between bacteria, including two Group I introns, an intein, and an intervening sequence (Seshadri et al., 2003; Raghavan et al., 2007, 2008; Beare et al., 2009; Warrier et al., 2016). Proliferation of mobile genetic elements and extensive genome rearrangements are hallmarks of bacteria that have recently shifted to host-associated lifestyles, indicating that the obligate intracellular growth of *C. burnetii* is of recent origin (Ochman and Moran, 2001; McCutcheon and Moran, 2012).

As expected, our analysis excluded several genes that were likely acquired via HGT because they are also present in other members of the order Legionellales e.g., TFSS genes, eukaryote-like sterol reductase genes, plasmid genes with eukaryotic domains etc. (Seshadri et al., 2003; Beare et al., 2009; Gilk et al., 2010; Voth et al., 2013). Without better resolution of *C. burnetii*'s phylogenetic position in the bacterial tree, it is difficult to discern whether these genes were acquired by a common ancestor or were gained independently by each bacterium as they adapted to their respective intracellular niches (Gottlieb et al., 2015). Intriguingly, *C. burnetii* encodes several intact or pseudogenized genes that encode the components of a Type IV pilus (Seshadri et al., 2003). A related Type IV pilus enables *Acinetobacter baumannii* to acquire DNA from the environment with high efficiency (Smith et al., 2007), indicating that during an earlier stage during *C. burnetii*'s evolution, a functional Type IV pilus endowed it with the ability to acquire foreign genes proficiently, thereby likely facilitating its transition into a mammalian pathogen from a tick-associated ancestor (Duron et al., 2015; Smith et al., 2015; Gerhart et al., 2016).

### HGT contributed to the lipopolysaccharide profile of *C. burnetii*

**Figure 2:**
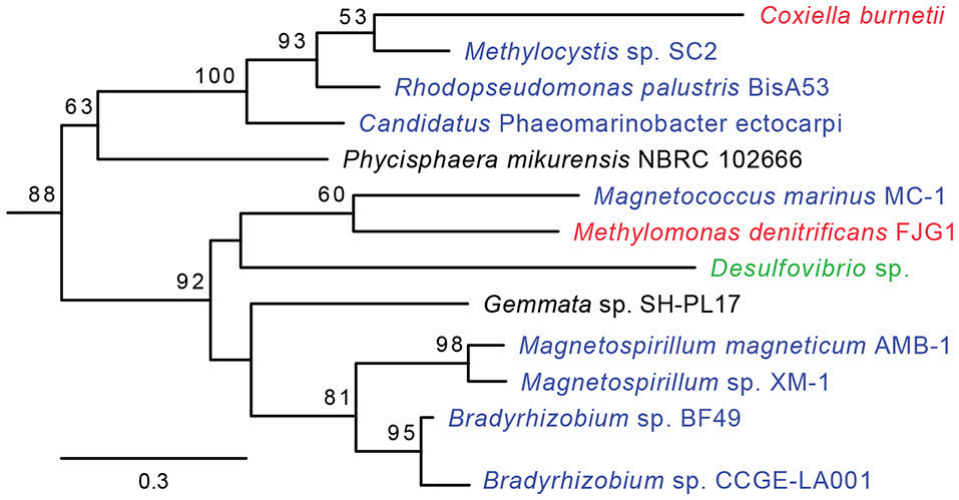
**CBU_0678 was gained via HGT**. A Maximum Likelihood phylogeny for CBU_0678, a LPS biosynthesis gene, is shown. Gammaproteobacteria are in red, Alphaproteobacteria in blue, Delta/Epsilonproteobacteria in green, and Planctomycetes in black. Bootstrap values of > 50 are indicated at the nodes.

HGT-origin genes are strewn all across the *C. burnetii* genome, indicating, as shown before, that there are no prototypical pathogenicity islands, but, based on their clustered genome locations, several genes appear to have been acquired en bloc (Figure 1). A group of genes of particular interest is CBU_0678 to CBU_0683, which is part of an operon that encodes genes involved in the biosynthesis of LPS (Seshadri et al., 2003; Narasaki and Toman, 2012). We conducted in-depth phylogenetic analyses of CBU_0678, the most upstream gene of this cluster, in order to validate the HGTector results. Based on both Maximum Likelihood and Bayesian phylogenetic analyses (Figure 2, Figure S1), the closest orthologs of this gene are present in members of Alphaproteobacteria. Furthermore, O-polysaccharide biosynthesis genes tend to occur as an operon in most bacteria, and since several genes in this location (CBU_0673, CBU_0676, CBU_0678 to CBU_0682) were also probably acquired from Alphaproteobacteria, it is highly likely that the genes were acquired in a single event. Previous studies have shown that LPS genes are horizontally transferred among bacteria (Nelson and Selander, 1994), probably due to their importance in host-pathogen interactions (Narasaki and Toman, 2012). In fact, full-length LPS is the only *C. burnetii* virulence factor established in an immunocompetent animal model of infection. It protects the pathogen from innate immune response (Shannon et al., 2005), and avirulent Nine Mile phase II produces a severely truncated LPS due to the loss of 22 LPS biosynthesis genes, including CBU_0678 to CBU_0682 (Beare et al., 2006). The observation that important virulence factors in *C. burnetii* (e.g., LPS, TFSS, effector proteins) were assembled via HGT illustrates the significance of this process in the evolutionary history of this intracellular pathogen.

### HGT enhanced *C. burnetii*'s fatty acid and biotin metabolism

**Figure 3:**
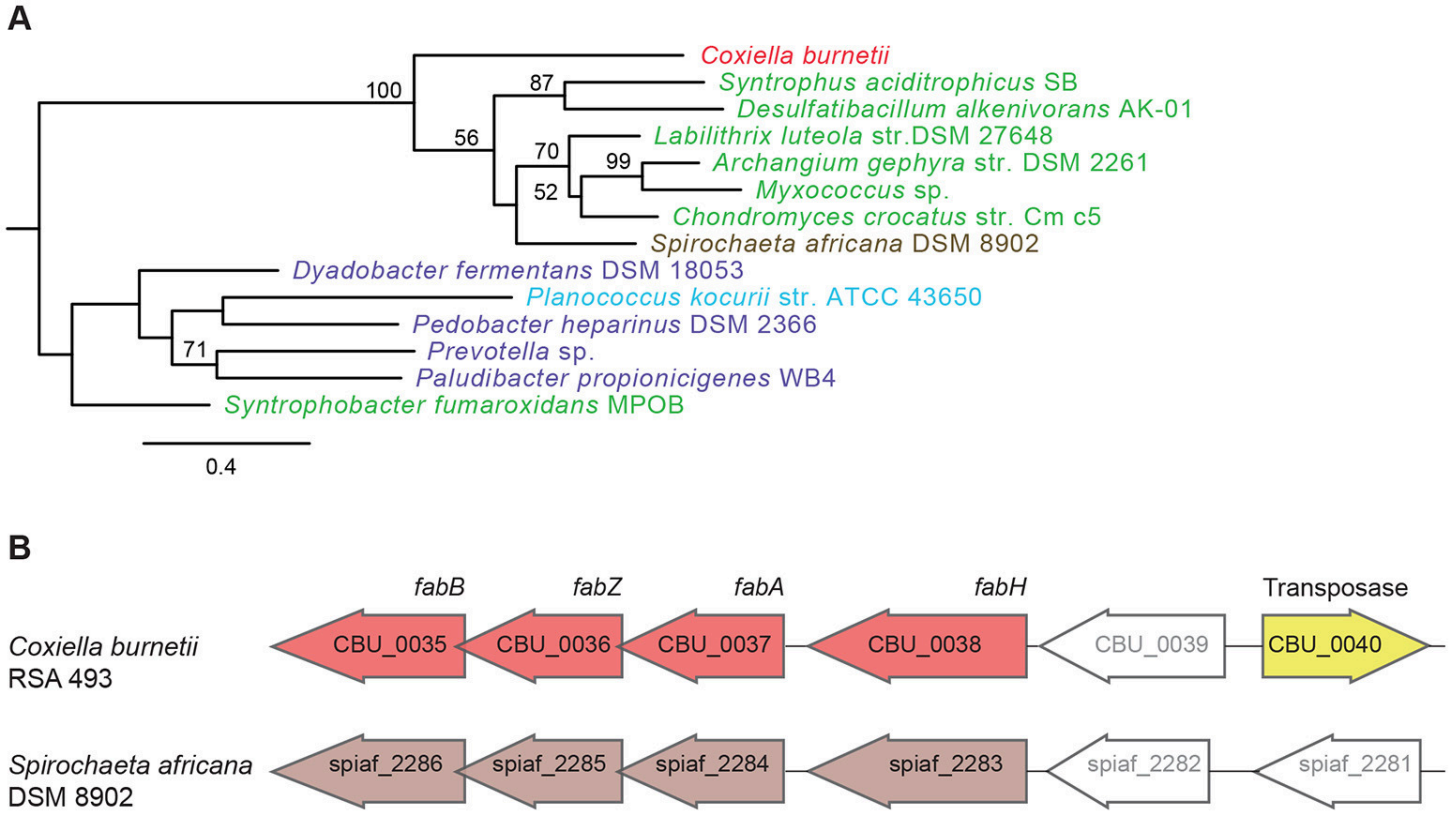
**A horizontally acquired fatty acid biosynthesis operon in ***C. burnetii***. (A)** Maximum Likelihood phylogenetic tree for CBU_0038, a fatty acid biosynthesis gene. Gammaproteobacteria is colored red, Delta/Epsilonproteobacteria in green, Spirochaetes in brown, Bacteriodetes in purple, and Firmicutes in blue. Bootstrap values of > 50 are indicated at the nodes. **(B)** Fatty acid biosynthesis genes have similar arrangement in *C. burnetii* and *Spirochaeta africana*, and an IS1111A transposase is located next to the operon in *C. burnetii*.

Another set of genes in *C. burnetii* that is of putative foreign origin is CBU_0034 to CBU_0038 (Figure 1). This gene cluster appears to have originated in either a Deltaproteobacteria or a Spirochete. We validated the HGT origin of this presumptive operon through phylogenetic analysis of its first gene, CBU_0038 (Figure 3A, Figure S2). Furthermore, this set of genes has a similar arrangement in both *C. burnetii* and in *Spirochaeta africana* DSM 8902, its best BLAST hit (Dataset S1), indicating that the genes were transferred en bloc. In addition, an IS1111A transposase (CBU_0040) is proximally located to the operon, illustrating a probable role for this mobile genetic element in HGT (Figure 3B). Based on homology, CBU_0034 to CBU_0038 encode ACP, FabB, FabZ, FabA, and FabH, respectively, which are involved in the synthesis of unsaturated fatty acids (Zhang and Rock, 2008; Feng and Cronan, 2009). In contrast, vertically inherited genes (CBU_0493 to CBU_0497) are responsible for the synthesis of saturated fatty acids (Gilk, 2012), denoting that *C. burnetii*'s ability to produce unsaturated fatty acids was enhanced through HGT. *C. burnetii* also contains a putative fatty acid desaturase (CBU_0920) that introduces double bonds into existing fatty acids (Gilk, 2012). This gene also appears to be of HGT origin, but was not included in our analysis because it is present in other Legionellales. In addition to fatty acid biosynthesis genes, *C. burnetii* has also gained three pyruvate dehydrogenases (CBU_0686, CBU_0692, and CBU_0693) (Figure 1). Pyruvate dehydrogenase complex converts pyruvate into acetyl-CoA, which is a critical metabolite for both fatty acid biosynthesis and ATP generation (de Kok et al., 1998). Thus, HGT has played a significant role in shaping *C. burnetii*'s fatty acid metabolism. Furthermore, a recent study showed that transposon-mediated disruption of CBU_0035 and CBU_0038 resulted in reduced *C. burnetii* growth within Vero cells, suggesting that this process is a critical constituent of the pathogen's physiology (Martinez et al., 2014).

**Figure 4:**
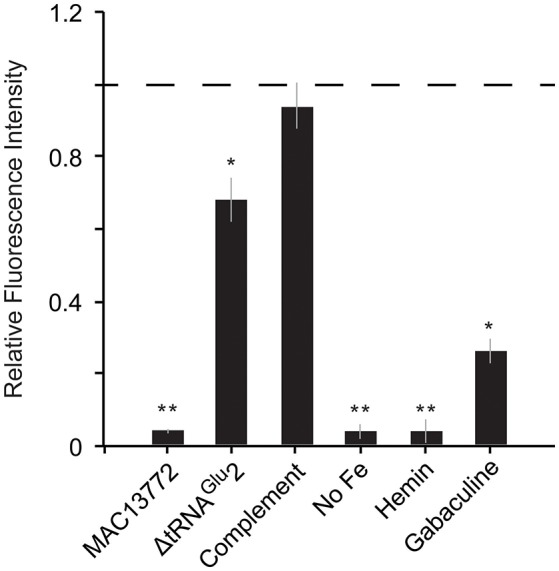
**Heme and biotin syntheses are critical to ***C. burnetii***'s growth**. Growth of *C. burnetii* in ACCM-2 after 7 days was measured using PicoGreen. Fluorescence of each strain relative to that of control (wild-type grown in ACCM-2; dashed line) is shown. **MAC13772**: ACCM-2 supplemented with 300 μg/ml MAC13772, a biotin biosynthesis inhibitor; Δ**tRNA**^Glu^**2**: tRNA^Glu^2-deletion strain; **Complement**: tRNA^Glu^2-deletion complemented with intact tRNA^Glu^2 on pAM100; **No Fe**: ACCM-2 without FeSO_4_; **Hemin**: ACCM-2 with hemin in place of FeSO_4_; **Gabaculine**: ACCM-2 supplemented with 100 μM gabaculine, a heme biosynthesis inhibitor. Statistically significant differences in growth from control are indicated by (^**^) *p* < 0.001 and (^*^) *p* < 0.01 (unpaired *t*-test).

Fatty acid biosynthesis enzymes require biotin as a cofactor, and biotin in turn is synthesized by utilizing a portion of the fatty acid biosynthesis pathway (Lin et al., 2010). Unlike most other bacteria, *C. burnetii* contains two copies of the gene *bioC*, the first committed step in biotin production. The two *bioC* genes are only 45% similar at the nucleotide level, indicating that they were not formed by a recent duplication event in *C. burnetii*. Further, *bioC.2* (CBU_1004) has an 11 bp overlap with *bioH* and is part of the *bioA-bioBFHCD-birA* regulon (CBU_1008 to CBU_1002), whereas *bioC.1* (CBU_0467) is a single gene located at a different part of the genome, indicating that *bioC1* is of horizontal origin. To understand the functional relevance of these genes, we examined their expression levels in *C. burnetii* grown in ACCM-2 and within Vero cells (Warrier et al., 2014). The RNA-seq data revealed that all biotin biosynthesis genes (*bioA, bioB, bioC.1, bioC.2, bioD, bioF*, and *bioH*) were expressed under both conditions, but their expression was significantly higher in Vero cells than in ACCM-2 (Table S1). In contrast, the expression of *birA*, the transcriptional repressor of biotin operon showed the opposite pattern, suggesting that biotin is likely synthesized under both conditions, with possible upregulation within the host cell. Furthermore, a small molecule (MAC13772) that blocks biotin biosynthesis in *E. coli* (Zlitni et al., 2013) inhibited the growth of *C. burnetii* in ACCM-2, indicating that the production of biotin is a critical process in this pathogen (Figure 4). Biotin synthesis has been shown to be critical to the virulence of other human pathogens such as *Francisella tularensis* and *Mycobacterium tuberculosis* (Woong Park et al., 2011; Feng et al., 2014). Thus, novel pharmaceutical agents that block this process hold promise as a potential broad-spectrum agent to treat intracellular pathogens.

### Heme biosynthesis is an essential metabolic process in *C. burnetii*

**Figure 5:**
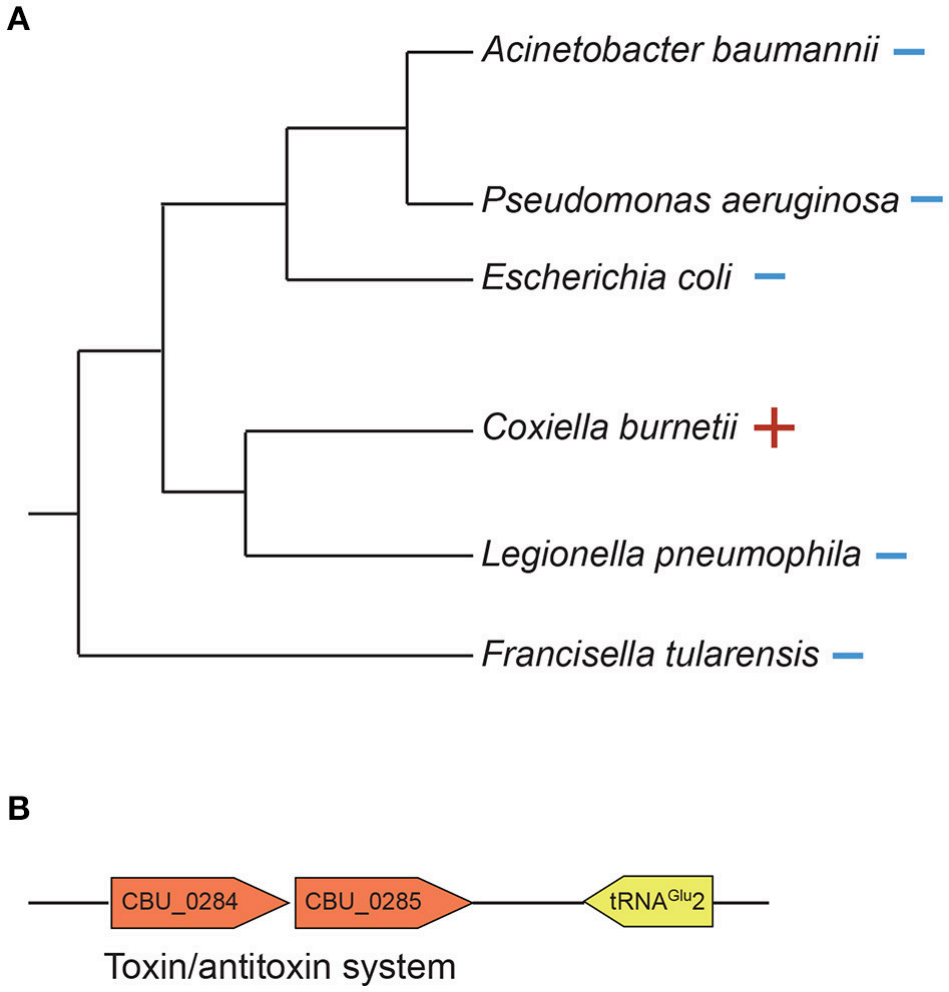
**tRNA^**Glu**^2 was horizontally acquired by ***C. burnetii***. (A)** 16S rDNA phylogenetic tree of Gammaproteobacteria showing the presence (+) or absence (–) of tRNA^Glu^(anticodon CUC). **(B)** A putative toxin/antitoxin system is located adjacent to tRNA^Glu^2.

*C. burnetii*'s genome is small compared to that of free-living bacteria such as *E. coli* (~2 and 5 Mb, respectively). Correspondingly, it contains less than half the number of tRNAs than in *E. coli* (42 and 89, respectively). This reduction has occurred due to loss of redundant tRNAs; for instance, while *E. coli* has five copies of tRNA^Ile^, *C. burnetii* has only one copy. In contrast to other tRNAs, *C. burnetii* contains an additional tRNA^Glu^ isoacceptor (tRNA^Glu^2, anticodon CUC) that is not found in any other Gammaproteobacteria, denoting that it was gained via HGT (Figure 5A). Additionally, a toxin-antitoxin system (CBU_0284, CBU0285) that was probably horizontally acquired is located adjacent to tRNA^Glu^2, further strengthening the evidence for its putative HGT origin (Figure 5B). Because tRNA^Glu^2 is retained in such a streamlined genome, it most likely has a critical function; in fact, a tRNA^Glu^2-deletion strain had significantly slower growth than the wild-type strain in ACCM-2, which was restored in a complementation strain, signifying the importance of the HGT-derived tRNA to optimum bacterial fitness (Figure 4). However, the major function of tRNA^Glu^2 is unlikely to be protein synthesis because tRNA^Glu^2 (anticodon CUC) cannot decode the major glutamate codon GAA (76%), whereas tRNA^Glu^1 (anticodon UUC) can decode both GAA and GAG codons efficiently. Additionally, *Coxiella* proteins are not enriched for glutamate in comparison to *E. coli* (~6% of all codons is for Glu in both bacteria). Taken together, these data suggest that protein biosynthesis is not tRNA^Glu^2's major function in *C. burnetii*.

An alternate function for tRNA^Glu^2 is in heme biosynthesis. Heme is an iron-containing tetrapyrrole that serves multiple cellular functions, including in respiration, energy generation, oxidative reactions, and signal transduction (Almiron et al., 2001). The universal precursor of tetrapyrrole biosynthesis is 5-aminolevulinic acid (ALA) (Anzaldi and Skaar, 2010). There are two alternate pathways in nature for the synthesis of ALA: C5 pathway found in most bacteria, and Shemin pathway present in most eukaryotes, including humans (Frankenberg et al., 2003). The starting point of the C5 pathway is Glutamyl-tRNA^Glu^, which is converted into ALA using two consecutive enzymes HemA and HemL. In Shemin pathway, ALA is synthesized from Succinyl-CoA and glycine by the enzyme ALAS (Frankenberg et al., 2003). The remaining steps are shared between the C5 and Shemin pathways. We examined the *C. burnetii* genome and discovered that it encodes an intact C5 heme biosynthesis pathway, and RNA-seq data confirmed that these genes are expressed during growth (Table S1). Additionally, heme biosynthesis pathway is conserved in all *C. burnetii* strains but is absent in non-pathogenic *Coxiella* present in ticks, and a *C. burnetii* strain with a transposon insertion in the *hemL* gene had reduced growth in Vero cells (Martinez et al., 2014), implying the importance of heme biosynthesis to the human pathogen.

Several bacteria augment heme biosynthesis with heme transported from the outside (Mike et al., 2013). *C. burnetii* encodes a transporter (*feoAB*) for ferrous iron, which is required for heme biosynthesis, but has no known transporters for ferric ions or heme. Consequently, we reasoned that the pathogen might not be able to utilize extracellular heme as its sole source of iron. To test this, we inoculated equal amounts of *Coxiella* into ACCM-2 medium with FeSO4 (standard recipe) or into ACCM-2 in which FeSO4 was replaced with equimolar amount of hemin. Growth was measured after 7 days using PicoGreen as described previously (Martinez et al., 2015). As shown in Figure 4, *Coxiella* growth in FeSO4-containing ACCM-2 was significantly higher than in hemin-containing ACCM-2 (*p* < 0.001, unpaired *t*-test), whereas the fluorescence measurements between the control (ACCM-2 without FeSO4) and the 1hemin-containing ACCM-2 samples were not significantly different (*p* > 0.05, unpaired *t*-test), showing that *C. burnetii* cannot utilize external heme. To test *C. burnetii*'s requirement for heme biosynthesis, we treated *C. burnetii* with increasing concentrations of gabaculine, an inhibitor of HemL (Wang et al., 1997), and found that 100 uM gabaculine significantly inhibited bacterial growth (*p* < 0.01, unpaired *t*-test) (Figure 4). These data show the importance of heme biosynthesis to *C. burnetii*'s normal physiology, and indicates that heme biosynthesis genes are potential targets for the development of new anti-*Coxiella* therapies.

In conclusion, by examining a few critical biosynthetic pathways we show that HGT has played an important role in shaping the metabolic capability of *C. burnetii*. Due to the uncertainty in *C. burnetii'*s phylogenetic relationship with other Gammaproteobacteria, we focused on a subset of genes that were likely acquired from distantly related bacteria. However, horizontal exchange usually occurs at higher frequency between closely related bacteria (Ochman et al., 2000), and hence, it is highly likely that an even larger proportion of genes in *C. burnetii* are of HGT origin. Availability of more genomes of bacteria in the phylogenetic neighborhood of *C. burnetii* along with more detailed evolutionary, genetic and functional analyses are required to identify all HGT-origin genes in *C. burnetii* and to fully understand their impact on the pathogen's physiology and pathogenicity.

## Materials and methods

### *C. burnetii* growth assay

*C. burnetii* Nine Mile phase II RSA 439 was grown in ACCM-2 medium as described previously (Omsland et al., 2011; Warrier et al., 2014), and incubated at 37°C, 2.5% O_2_, and 5.0% CO_2_ using a Galaxy 170 R incubator (New Brunswick Scientific, NJ). Chloramphenicol (8 μg/ml) and/or Kanamycin (375 μg/ml) were added as necessary. Growth was measured using PicoGreen as described previously (Martinez et al., 2015). Briefly, 50 μl of culture was mixed with 5 μl of Triton X-100 Surfact-Amps 10% detergent solution (Thermo Scientific) in 96-well black-bottom Cliniplates (Thermo Scientific), and allowed to incubate at room temperature for 10 min with shaking. PicoGreen (Life Technologies) was diluted 1:200 in TE buffer and 55 μl was added to the wells, and incubated at room temperature with shaking for 5 min. Wells were excited at 495 nm and emission was read at 519 nm using a Victor X5 2030 Multiplate Reader (Perkin Elmer). To determine whether *Coxiella* can utilize external heme as its sole iron source, ferrous sulfate was either omitted from ACCM-2 preparations or substituted with hemin (Alfa Aesar), and to assay the importance of heme and biotin biosyntheses, gabaculine (Enzo Life Sciences) or MAC13772 (Maybridge) was added to ACCM-2. Briefly, a 10 mM stock of hemin was made in 1.5 M NaOH, a 300 mg/ml stock of MAC13772 was made in dimethyl sulfoxide (DMSO), and a 100 mM stock of gabaculine was made in distilled water. One microliter of solution from these stocks was added to 1 ml of ACCM-2 to attain final concentrations of 10 μM hemin, 300 μg/ml MAC13772, or 100 μM gabaculine. ACCM-2 containing the same amount of solvent was used as control, and growth was measured after 7 days using PicoGreen as described above.

### Generation of tRNA^Glu^2 deletion and complementation strains

To delete tRNA^Glu^2, ~1200 bp on each side of the gene along with Chloramphenicol acetyltransferase (CAT) gene was cloned into the vector pJC-Kan, and the mutant was generated as described previously (Beare et al., 2012). Insertion of the CAT gene in place of tRNA^Glu^2 was confirmed using PCR and DNA sequencing. To generate a complementation strain we used pKM244, a low-copy plasmid that is maintained stably in *C. burnetii* (Chen et al., 2010). Because the tRNA^Glu^2-deletion strain already contained a CAT gene, we amplified a kanamycin resistance gene driven by 1169^P^ from pJB-Kan (Beare et al., 2012) and cloned it into pKM244 using NheI and AatII to generate pAM100. We inserted tRNA^Glu^2 along with its flanking intergenic regions into pAM100 using BamHI. *C. burnetii* was transformed (400 ohms, 2.5 kV, 25 mF) with either empty pAM100 or pAM100 with cloned tRNA^Glu^2, as described previously (Omsland et al., 2011; Beare et al., 2012). Growth in ACCM-2 of wild-type (with empty pAM100), deletion (with empty pAM100) and complementation strains were measured at day-7 using PicoGreen as described above.

### Detection of horizontal gene transfer

Horizontally acquired genes were identified using HGTector (Zhu et al., 2014). *Coxiella* was set as self-group, and Legionellales was set as exclusion group. BLAST parameter thresholds were set at 70% identity and an E-value of at least 1e-5. To validate the HGT data, phylogenetic analyses were conducted for several putative HGT genes. To this end, TBLASTN search was conducted against NCBI nr database and the top 100 matches with at least 30% identity, 70% coverage, and E-value of 1e-10 were chosen. Sequence alignment was performed using Clustal Omega (Sievers et al., 2011), and ambiguously aligned regions were removed using Gblocks (Talavera and Castresana, 2007). The evolution model GTR+I+G (General Time Reversible plus Invariant sites plus Gamma distribution) was selected using jModelTest2 (Darriba et al., 2012). Bayesian trees were constructed using MrBayes as implemented in Geneious (Huelsenbeck and Ronquist, 2001; Kearse et al., 2012). A chain length of 1,000,000 was used with a burn-in fraction of 25% and sampling every 100 trees. Maximum Likelihood trees were constructed using RAxML (Stamatakis et al., 2008) as implemented in Geneious with 1,000 bootstrap replicates.

## Author contributions

AM, JM, MB, PB, and RR conducted the experiments, analyzed the data, and prepared the manuscript.

### Conflict of interest statement

The authors declare that the research was conducted in the absence of any commercial or financial relationships that could be construed as a potential conflict of interest.
