# Supplementary material for: Horizontally Acquired Biosynthesis Genes Boost Coxiella burnetii's Physiology
Source: Front Cell Infect Microbiol. 2017 May 10;7:174. doi: 10.3389/fcimb.2017.00174 (PMC5423948; doi:10.3389/fcimb.2017.00174)
Supplement: Supplementary file 3 [file Table1.DOCX]

**Table S1.** Expression of biotin and heme biosyntheses genes in *C. burnetii* grown in ACCM-2 or Vero cells for 72 h measured using RNA-seq.

| **Gene** | **ACCM-2 (LCV)** | **Vero (LCV)** | **Fold Change** | ***p*-value**  **(FDR corrected)** |
| --- | --- | --- | --- | --- |
| **Biotin** |  |  |  |  |
| *bioA* | 2,093 | 3,915 | 1.87 | <0.001 |
| *bioB* | 1,562 | 10,118 | 6.48 | <0.001 |
| *bioC.1* | 9,659 | 15,678 | 1.62 | <0.001 |
| *bioC.2* | 1,562 | 2,909 | 1.86 | <0.001 |
| *bioD* | 1,590 | 6,233 | 3.92 | <0.001 |
| *bioF* | 1,159 | 5,354 | 4.62 | <0.001 |
| *bioH* | 916 | 4,836 | 5.28 | <0.001 |
| *birA* | 5,803 | 3,961 | 0.68 | 0.0055 |
| **Heme** |  |  |  |  |
| *hemA* | 6,447 | 10,335 | 1.60 | 0.0007 |
| *hemB* | 49,632 | 5,778 | 0.12 | <0.001 |
| *hemC* | 6,652 | 4,783 | 0.72 | 0.0183 |
| *hemD* | 4,534 | 6,420 | 1.42 | 0.0110 |
| *hemE* | 29,442 | 16,762 | 0.57 | <0.001 |
| *hemF* | 9,104 | 12,200 | 1.34 | 0.0291 |
| *hemH* | 3,343 | 8,085 | 2.42 | <0.001 |
| *hemK* | 3,497 | 6,141 | 1.76 | <0.001 |
