# Supplementary material for: Horizontally Acquired Biosynthesis Genes Boost Coxiella burnetii's Physiology
Source: Front Cell Infect Microbiol. 2017 May 10;7:174. doi: 10.3389/fcimb.2017.00174 (PMC5423948; doi:10.3389/fcimb.2017.00174)
Supplement: Supplementary file 2 [file Image2.PDF]

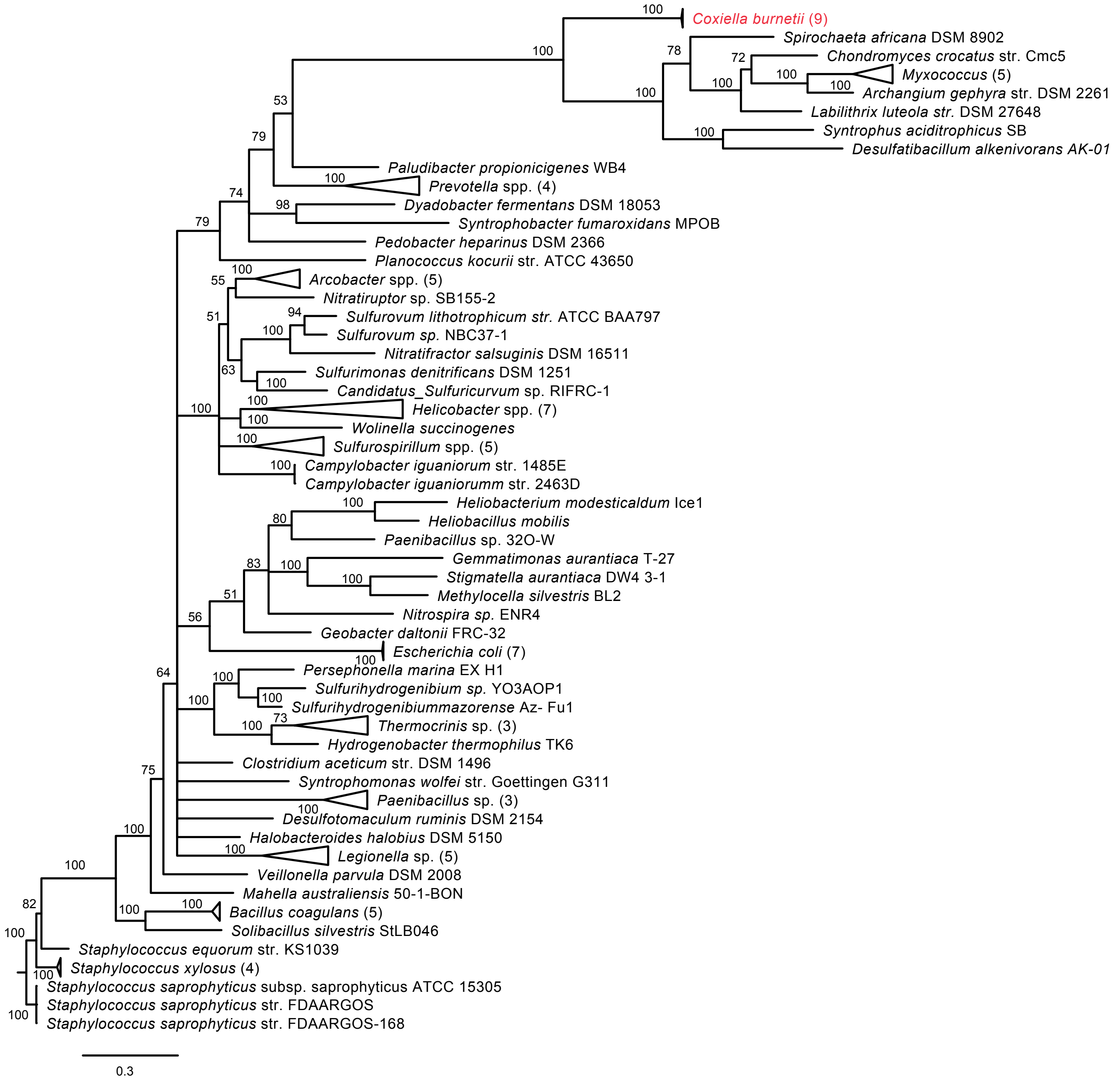

Figure S2. CBU_0038 is of HGT origin. A Bayesian phylogenetic tree of CBU_0038. Posterior probability shown at nodes. *C. burnetii* highlighted in red. Number of taxa collapsed into each branch is shown within parentheses.
